# Phenotypic and Genomic Analyses of *Burkholderia stabilis* Clinical Contamination, Switzerland 

**DOI:** 10.3201/eid2506.172119

**Published:** 2019-06

**Authors:** Helena M.B. Seth-Smith, Carlo Casanova, Rami Sommerstein, Dominik M. Meinel, Mohamed M.H. Abdelbary, Dominique S. Blanc, Sara Droz, Urs Führer, Reto Lienhard, Claudia Lang, Olivier Dubuis, Matthias Schlegel, Andreas Widmer, Peter M. Keller, Jonas Marschall, Adrian Egli

**Affiliations:** University Hospital Basel, Basel, Switzerland (H.M.B. Seth-Smith, D.M. Meinel, A. Widmer, A. Egli);; University of Basel, Basel (H.M.B. Seth-Smith, D.M. Meinel, A. Egli);; University of Bern, Bern, Switzerland (C. Casanova, S. Droz);; Bern University Hospital, Bern (R. Sommerstein, J. Marschall);; Lausanne University Hospital, Lausanne, Switzerland (M.M.H. Abdelbary, D.S. Blanc);; Biel Hospital, Biel, Switzerland (U. Führer);; ADMED, La Chaux-de-Fonds, Switzerland (R. Lienhard);; Viollier AG, Allschwil, Switzerland (C. Lang, O. Dubuis);; Cantonal Hospital St. Gallen, St. Gallen, Switzerland (M. Schlegel);; Swissnoso, National Center for Infection Prevention, Bern (M. Schlegel, A. Widmer, J. Marschall);; University of Zurich, Zurich, Switzerland (P.M. Keller)

**Keywords:** bacteria, Bcc, *Burkholderia stabilis*, DNA, resistance, virulence, whole-genome sequencing, hospital-associated infections, Switzerland

## Abstract

A recent hospital outbreak related to premoistened gloves used to wash patients exposed the difficulties of defining *Burkholderia* species in clinical settings. The outbreak strain displayed key *B. stabilis* phenotypes, including the inability to grow at 42°C; we used whole-genome sequencing to confirm the pathogen was *B. stabilis*. The outbreak strain genome comprises 3 chromosomes and a plasmid, sharing an average nucleotide identity of 98.4% with *B. stabilis* ATCC27515 BAA-67, but with 13% novel coding sequences. The genome lacks identifiable virulence factors and has no apparent increase in encoded antimicrobial drug resistance, few insertion sequences, and few pseudogenes, suggesting this outbreak was an opportunistic infection by an environmental strain not adapted to human pathogenicity. The diversity among outbreak isolates (22 from patients and 16 from washing gloves) is only 6 single-nucleotide polymorphisms, although the genome remains plastic, with large elements stochastically lost from outbreak isolates.

*Burkholderia* is a diverse genus of gram-negative bacteria, with isolates identified from a variety of environments, and ever more species being identified and classified. Whereas some *Burkholderia* species are associated with bioremediation potential and antimicrobial and antifungal production, others are animal and human pathogens that generally fall within the *B. cepacia* complex (Bcc) (*1*). *Burkholderia* bacteria have large, flexible, multi-replicon genomes, a large metabolic repertoire, various virulence factors, and inherent resistance to many antimicrobial drugs (*2*,*3*).

An outbreak of *B. stabilis* was identified among hospitalized patients across several cantons in Switzerland during 2015–2016 (*4*). The bacterium caused bloodstream infections, noninvasive infections, and wound contaminations. The source of the infection was traced to contaminated commercially available, premoistened washing gloves used for bedridden patients. After hospitals discontinued use of these gloves, the outbreak resolved. 

Many instances of Bcc strain contamination of medical devices and solutions have been described (*4*), including an outbreak in Korea associated with a 0.5% chlorhexidine solution (*5*). *B. stabilis* also has been identified in nosocomial infections (*6*–*8*).

We conducted in-depth characterization of the *B. stabilis* strain from the Switzerland outbreak by using clinical methods and whole-genome sequencing (WGS). We generated a complete draft genome by combining short- and long-read genomic data and compared it to other outbreak isolates to provide a complete genomic assessment of this strain. We provide a thorough comparative genomic analysis of this outbreak strain.

## Methods

### Bacterial Isolate Collection

Isolates were collected from 22 patients (labeled 1–22) and 16 contaminated washing gloves (labeled A–P) across Switzerland during the outbreak (*4*). For comparison, we collected 14 unrelated *Burkholderia* spp. patient isolates in Switzerland (labeled O-1 through O-14; Appendix 1 Table 1). We obtained a control strain, *B. stabilis* ATCC27515 BAA-67, isolated in 1993 from sputum of a patient with cystic fibrosis in Belgium, from the American Type Culture Collection (ATCC, https://www.atcc.org).

### Clinical Diagnostics

We performed routine identification using matrix-assisted laser desorption/ionization time-of-flight (MALDI-TOF) mass spectrometry and biochemical species identification. For MALDI-TOF mass spectrometry, we used Biotyper MBT Smart (Bruker Corporation, https://www.bruker.com) with flexControl and MBT Compass version 4.1 software. We considered scores >2.0 high confidence identification and scores of 1.7–2.0 low confidence identification. We used VITEK 2 gram negative identification card (bioMérieux, https://www.biomerieux.com) for biochemical species identification. Phenotypic antimicrobial resistance profiles were determined using disk diffusion. We interpreted breakpoints according to Clinical and Laboratory Standards Institute (*9*) standards for Bcc (ceftazidime, trimethoprim/sulfamethoxazole, and meropenem) or *Enterobacteriaceae* (aminoglycosides, ciprofloxacin, piperacillin/tazobactam, and other β-lactams). We used *Xba*I to digest DNA before using previously described pulsed-field gel electrophoresis (PFGE) molecular typing principles (*10*). We used GelCompar (Applied Maths, http://www.applied-maths.com) to analyze PFGE results.

### Cellular Fatty Acid Analysis

We prepared and derivatized cellular fatty acids from outbreak isolates 7, 13, and O, with control strain *Pseudomonas aeruginosa* strain ATCC27853, as previously described (*11*). We performed chromatography on an HP 6890 gas chromatograph (Hewlett Packard Enterprise, https://www.hpe.com) and analyzed data in SHERLOCK MIS version 6.2 (Midi Inc., http://midi-inc.com).

### Genome Sequencing, Assembly, Annotation, and Mapping

We extracted DNA using EZ1 Advanced XL (QIAGEN, https://www.qiagen.com) or Wizard Genomic DNA Purification kit (Promega, https://www.promega.com) and then sequenced it on the Illumina MiSeq platform (https://www.illumina.com) following Nextera XT library creation within the Division of Clinical Microbiology, University Hospital Basel (300-bp paired-end reads) or the Unit of Genomics of the Institute of Microbiology, Lausanne University Hospital (150-bp paired-end reads). We mapped data against the genome of *B. cepacia* ATCC25416 (GenBank accession nos. CP007746–8) for quality control and coverage determination (Appendix 1 Table 1). We sequenced DNA from outbreak isolate 5 on a PacBio RS II platform (Pacific Biosciences, https://www.pacb.com) with 1 SMRT cell at the Functional Genomics Centre, Zurich. We submitted read data for all samples to the European Nucleotide Archive under project nos. PRJEB18658 and PRJEB19203 (data previously analyzed; *12*). 

We used CLC Genomics Workbench 9 (https://clc-genomics-workbench.software.informer.com/9.0) to assemble Illumina reads from outbreak isolate E (1,736 contigs; assembly length 8,816,302 bp) and from unrelated isolates. We processed PacBio reads with CLC Genomics Workbench 9 using an error correction of 30 or 50 and assembled resulting reads or used them to correct the Illumina assembly of E. We used Spades version 3.10.0 (*13*) to assemble the PacBio reads with Illumina reads from isolates E or 5. We manually compared assemblies in Artemis and ACT (*14*,*15*) to circularize and split chromosomes 1 and 2 according to the genome of *B. stabilis* BAA-67 (GenBank accession nos. CP016442–4) (*16*). We found chromosome 3 is a single contig that was not obviously circularizable. We used Prokka version 1.11 (*17*) for automated annotation and manually curated coding sequences (CDSs) by using Artemis and ACT. We submitted the genome draft to ENA under accession no. ERZ480954.

We performed mapping in CLC Genomics Workbench 9, which we also used to generate k-mer trees (*18*) using default parameters. For single-nucleotide polymorphism (SNP) phylogenies, we used variant calling with 5× minimum coverage, 5 minimum count, and 70% minimum frequency and created SNP trees with 5× minimum coverage, 5% minimum coverage, 0 prune distance, and multinucleotide variants.

### Database and Genome Comparisons

We used multilocus sequence typing (MLST; https://cge.cbs.dtu.dk/services/MLST) to identify alleles from assemblies of isolate E and unrelated *Burkholderia* spp. (*19*) and compared these against Bcc MLST databases (https://pubmlst.org/bcc) (*20*) for species designation. We performed average nucleotide identity (ANI) determination using the ANI calculator (http://enve-omics.ce.gatech.edu/ani) (*21*), and digital DNA-DNA hybridization (dDDH) with GGDC2.1 (http://ggdc.dsmz.de/distcalc2.php) (*22*). We used antiSMASH version 4.0.0 (https://antismash.secondarymetabolites.org) (*23*) to predict gene clusters involved in antimicrobial resistance and secondary metabolite production.

## Results

### Clinical and Major Fatty Acid Characterization

**Figure 1:**
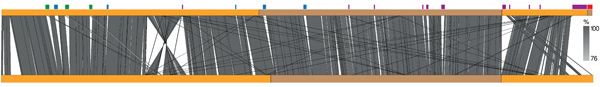
Comparison of the genome of *Burkholderia stabilis* strain CH16 from Switzerland (top bar) with that of *B. stabilis* reference strain BAA-67 (bottom bar). Alternating orange and brown bar sections represent chromosomes 1, 2, 3, and a plasmid. Scale bar indicates identity between the genomes (determined by blastn, http://blast.ncbi.nlm.nih.gov). Colors above the CH16 genome indicate the following: purple, regions of difference between the 2 strains; green, putative integrative and conjugative element; blue, phage; and red, the plasmid.

We analyzed outbreak and unrelated *Burkholderia* spp. isolates by PFGE (Appendix 2 Figure 1). PFGE patterns of outbreak isolates from patients and washing gloves formed a cluster separate from the unrelated isolates. Outbreak isolates shared 75.7% similarity by Pearson correlation analysis; previous studies used a value of 80% for outbreak grouping (*24*).

We conducted MALDI-TOF mass spectrometry on 12 outbreak isolates and identified *B. cepacia* group (n = 4), *B*. *stabilis* (n = 4), *B. multivorans* (n = 2), *B. cenocepacia* (n = 1), and *B. pyrrocinia* (n = 1) with scores of 1.88–2.21. Because MALDI-TOF mass spectrometry is known to misidentify Bcc species (*25*), we used VITEK 2 to conduct biochemical testing on isolates from 3 patients and 1 washing glove and identified either Bcc group (scores of 91%–95%) or *Acinetobacter lwoffii* (scores of 86%–91%). 

Our outbreak isolates and control strain shared a defining characteristic of *B. stabilis*, the inability to grow at 42°C (*26**,**27*). Using VITEK 2, we saw 2 other key characteristics of *B. stabilis* in the outbreak strain, absence of β-galactosidase activity and inability to oxidize sucrose (Table 1). In contrast to other *B. stabilis* strains, VITEK 2 showed our strain was negative for adonitol acidification, ornithine decarboxylase, and lysine decarboxylase. Phenotypic identification of *Burkholderia* spp. often is a tedious process (*26*), with high rates of misidentification because of false negative reporting by VITEK 2 (*29*). Clinical standard identification on the VITEK 2 runs up to 16 hours, but *Burkholderia* phenotypes can take up to 7 days to develop. Our subsequent genome analysis identified genes encoding ornithine decarboxylase and lysine decarboxylase in the outbreak strain.

**Table 1:** Biochemistry of *Burkholderia stabilis* outbreak strain from 3 patient isolates and 1 environmental isolate collected from hospitals in Switzerland, 2015–2016*

Reaction	Result	No. outbreak strains with result	Expected *B. stabilis* result†
Saccharose, sucrose‡	–	4	–
β-galactosidase‡	–	4	–
Maltose acidification	+	3	+
Adonitol acidification	–	4	+
Ornithine decarboxylase	–	4	+
Lysine decarboxylase	–	4	+
D-mannitol	–	4	+
D-glucose	+	4	+
D-cellobiose	+	4	+
Malonate	–	4	+
D-sorbitol	–	4	+
Urease	–	4	+ or –

*Performed using VITEK 2.  †From (*26,28*). ‡Specific discriminatory test for *B. stabilis*.

**Figure 2:**
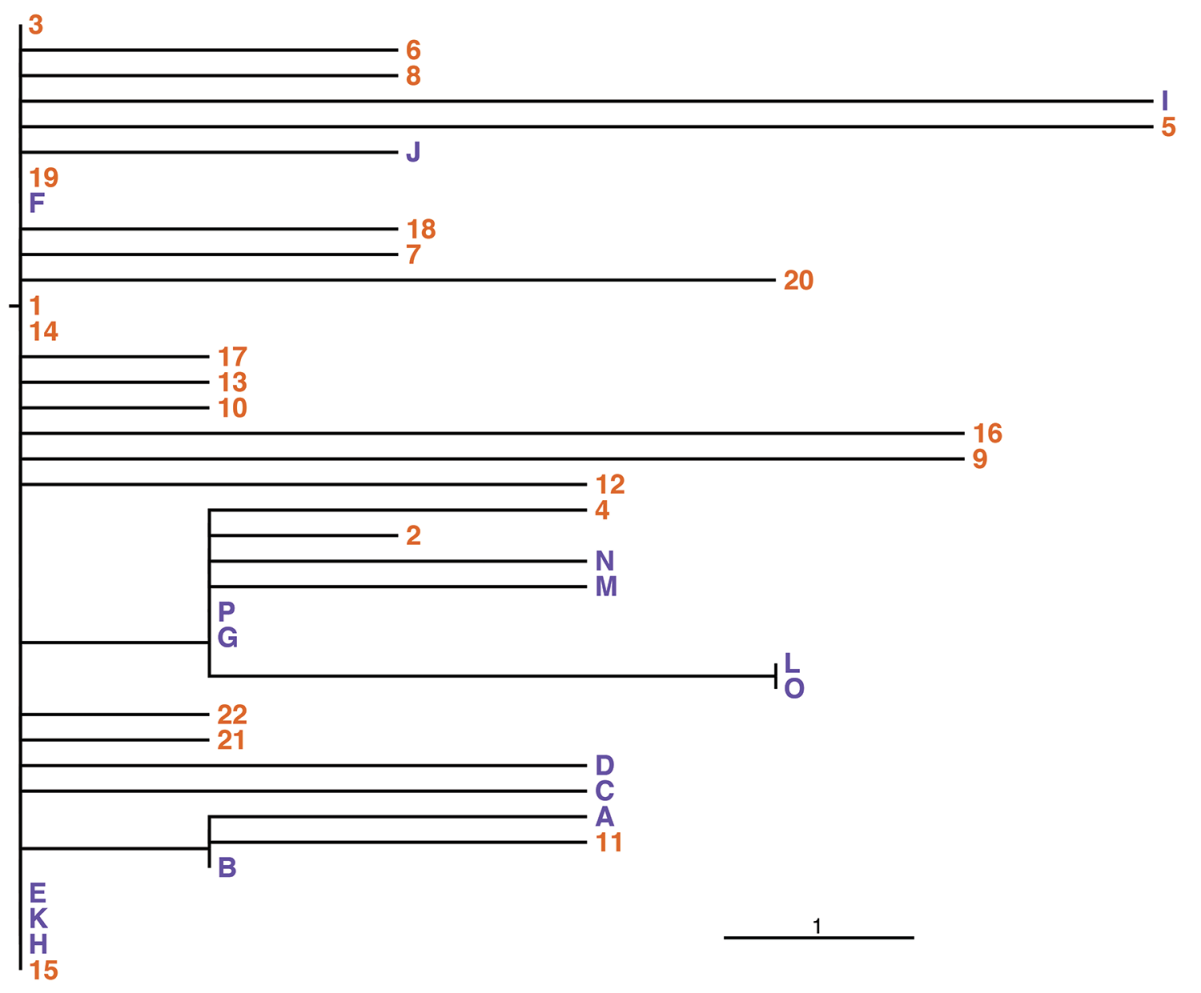
Phylogeny of outbreak isolates of *Burkholderia stabilis* strain CH16 from Switzerland based on high-quality single nucleotide polymorphisms (SNPs). This phylogeny of all sequenced outbreak isolates might represent a conservative estimate of SNP numbers. Given the large genome size and possible mismapping to repeats, it is difficult to determine the ultimate number of SNPs between samples. This phylogeny was confirmed using several parameters and manual checking of called SNPs. The root was arbitrarily chosen to give the fewest root to tip SNPs (n = 6). Numbers represent isolates from patients; letters represent isolates from washing gloves, located in the root position. Scale bar indicates 1 SNP.

Cellular fatty acid profiling of 3 outbreak isolates showed profiles highly similar to the reference strains *B. stabilis* and *B. cepacia* (*26*,*30*), and expectedly distinct from control strain *P. aeruginosa* strain ATCC27853 (Appendix 1 Table 2; Appendix 2 Figure 2). Together, these assays identified the outbreak strain as a member of the genus *Burkholderia* within Bcc but did not enable a firm species-level identification.

### Identification of Outbreak Isolate Clade in Bcc

We conducted WGS on 22 patient isolates and 16 isolates from washing gloves. We compared a full-length 16S rRNA gene sequence derived from the genome assembly of isolate E against the National Center for Biotechnology Information nucleotide sequence database using blastn (http://blast.ncbi.nlm.nih.gov). We identified 2 top hits, both sharing 1,520 out of 1,521 nt identities, *B. pyrrocinia* DSM10685, and *B. stabilis* BAA-67. Many other Bcc species shared >99% nucleotide identity, including *B. stagnalis* MSMB735WGS, *B. cenocepacia* AU1054, *B. cenocepacia* J2315, *B. ambifaria* AMMD, and *B. lata* 383. We mapped the WGS data of all outbreak isolates against a draft assembly of isolate E and found 99%–100% coverage from all isolates, but only 60%–75% coverage from unrelated isolate sequences (Appendix 1 Table 1). K-mer analysis of outbreak and unrelated *Burkholderia* isolates showed a separate cluster of outbreak isolates (Appendix 2 Figure 3).

### Identification of New *B. stabilis* Strain

We used MLST to extract alleles from the genome draft to obtain a new sequence type (ST), 1095, with the following genes: *atpD* (380, new), *gltB* (456), *gyrB* (213), *lepA* (70), *phaC* (348, new), *recA* (109), *trpB* (173). The top matches to this MLST profile are all *B. stabilis* isolates sharing <4 of the 7 alleles. The matching isolates are from Canada, which had 4 matching alleles; and Czechia, Serbia, China, France, Italy, and the United Kingdom, each with 2 matching alleles, including the *B. stabilis* type strain ATCC27515 BAA-67.

We used ANI calculations of genomic relatedness to compare the isolate E genome draft to a comprehensive panel of sequenced Bcc strains (Table 2). *B. stabilis* BAA-67 is the most closely related with an ANI of 98.4%, which is above the species cutoff of 95% (*31*). In addition, dDDH comparing the outbreak strain to the Bcc panel showed that the maximum score of the outbreak genome is with *B. stabilis* BAA-67 at 84.2%, with the classic species threshold at 70%. These genomic parameters currently are the most robust for species designation (*32*–*34*) and we are confident that the outbreak strain belongs to the species *B. stabilis*. The high dDDH score might reflect the high genome conservation within this species, giving it its name (*26*).

**Table 2:** Genome comparisons of *Burkholderia stabilis* outbreak strain CH16 from Switzerland against *Burkholderia* reference strains*

Reference genome	Genomovar	GenBank accession nos.	ANI,† 1-way,%	dDDH,‡ formula 2	Probability dDDH >70%	% G+C difference
*B. stabilis* ATCC BAA-67	IV	CP016442–4	98.4	87.8	94.98	0.08
*B. pyrrocinina* DSM10685	IX	CP011503–6	92.9	49.4	17.44	0.17
*B. stabilis* LA20W§	IV	GCA_001685505.1	92.5	49.3	17.3	0.16
*B. lata* 383		NC_007509–11	91.4	44.7	7.72	0.12
*B. cepacia* ATCC25416	I	NZ_CP012981–3	91.3	44.5	7.52	0.37
*B. cenocepacia* J2315	III	AM747720–3	91.1	44	6.68	0.55
*B. ambifaria* AMMD	VII	NZ_CP009798–800	89.8	39.9	2.65	0.44
*B. latens* AU17928		CP013435–8	88.8	37	1.22	0.03
*B. dolosa* AU0158	VI	CP009793–5	88.8	37.2	1.27	0.66
*B. vietnamensis* LMG10929	V	CP009629–32	88.5	36.2	0.97	0.48
*B. multivorans* DDS 15A-1	II	CP008728–30	88.3	36	0.89	0.25
*B. multivorans* BAA247	II	CP009830–2	88.1	35.3	0.71	0.9

*ANI, average nucleotide identity; dDDH, digital DNA-DNA hybridization. †Species cutoff 95%.  ‡Species cutoff 70%. §From these results, strain LA20W should not be classified as *B. stabilis*.

### Description of Draft of *B. stabilis* Strain CH16

A hybrid assembly of PacBio and Illumina data resulted in an improved, high-quality genome draft (*35*) of the outbreak strain, named CH16 because it occurred in Switzerland in 2016. This draft comprises 1 contig for each of the 3 chromosomes. Comparison with the genome of *B. stabilis* BAA-67 (*16*) showed that the genomes are syntenic with the exception of a rearrangement on chromosome 1 between the rRNA operons, which might be a real inversion or an assembly artifact in 1 of the genomes (Figure 1). We detected a separate contig representing a predicted plasmid sequence, whereas none was found within strain BAA-67 (J. Bugrysheva, US Centers for Disease Control and Prevention, pers. comm., 2017 Jan 10) (Table 3).

**Table 3:** General properties of genome draft for *Burkholderia stabilis* strain CH16 from Switzerland*

Property	Value
Draft genome size, bp	8,505,958
Chromosomes	3
Chromosome sizes, bp	3,705,321; 3,499,410; 1,230,432
Plasmid size, bp	70,922
% G+C content	66.3
Predicted CDSs (per chromosome and plasmid)	7,629 (3,402; 3,068; 1,075; 84)
Coding density	86.40%
Average gene length	965 bp
Pseudogenes	20
rRNA operons	6
tRNAs	75
Insertion elements	40

*CDSs, coding sequences.

In addition to being large and multireplicon, *Burkholderia* genomes are characterized by the presence of multiple phages, genomic islands, and insertion sequences (IS elements) (*3*). The draft genome CH16 contains many insertions of single or multiple genes relative to strain BAA-67: 16 on chromosome 1; 34 on chromosome 2; and 10 on chromosome 3 (Figure 1). Appendix 1 Table 3 lists regions of difference (RDs).

The *B. stabilis* CH16 genome has a paucity of IS elements. We have identified only 40, including 6 families with copy numbers of 3–12 (Appendix 1 Table 4), that cause disruption of 9 CDSs (Appendix 1 Table 5). CH16 did not appear to be experiencing IS element expansion, which is associated with genome rearrangements, large-scale genomic deletions, and niche adaptation (*36*–*38*), but it has the potential for IS element expansion if it goes through a population bottleneck. 

Frameshifts and premature stop codons have created 11 additional pseudogenes (Appendix 1 Table 5). The 20 pseudogenes of CH16 contrast with 142 annotated in the genome of strain BAA-67, indicating that most of the CH16 genome is required for survival in diverse environments and that this strain is not adapting to a pathogenic lifestyle.

### RDs and Virulence Factors of *B. stabilis* Strain CH16

Using genome-wide blastn comparisons, we determined that the CH16 genome carries 973 novel CDSs relative to BAA-67 of the total 7,629 CDSs (12.7%; Appendix 1 Table 3), many of which are novel to all *Burkholderia* sequenced to date. Larger insertions containing >40 CDSs are putative phages or integrative and conjugative elements. Smaller insertions of <10 CDSs appear to represent deletions in the BAA-67 strain relative to their common ancestor.

Factors that might contribute to the virulence of CH16 include adhesins and hemaglutinins, including *BSTAB16_1184*, *_5825*, *_5829*, *_5874*, *_6110*, *_6684*, *_6804*, and _*6861*, of which most have homologs in other Bcc strains; and Type II and Type VI secretion systems (*BSTAB16_4657–74*, _*5069–91*, and _*5583–9*). The many regulators within the CH16 genome and the RDs provide additional layers of translational control necessary in a genome of this size. We saw no evidence of the known *Burkholderia* virulence factors cable pilus or *B. cepacia* epidemic strain marker (*36*). The toxins we identified, for example *BSTAB16_5843* containing the HipA domain, are antibacterial toxins rather than virulence factors.

The many efflux pumps found in the CH16 genome might explain its ability to grow in the wash solution, including members of the following families: resistance nodulation and cell division, ATP-binding cassette, small multidrug resistance, multidrug and toxic compound extrusion, and major facilitator superfamily. Several secondary metabolite synthesis pathways are predicted: 4 on chromosome 1; 5 on chromosome 2; and 5 on chromosome 3 (Appendix 1 Table 6). Most of these are shared with the BAA-67 reference genome, encoding the ability to produce signaling molecules, siderophores, terpenes, and a bacteriocin, among others.

The plasmid comprises largely novel sequences not seen before within the *Burkholderia* or elsewhere. It carries genes predicted to be involved in conjugation, indicating that it might be a mobile plasmid, such as 1 recently hypothesized in *B. cenocepacia* (*39*). The rest of the plasmid largely comprises genes encoding hypothetical proteins.

### Antimicrobial Drug Resistance of *B. stabilis* Strain CH16

We performed phenotypic antimicrobial drug susceptibility testing on a subset of outbreak isolates (Appendix 1 Table 7) and used genomic findings to interpret the results. Breakpoints are not established clinically and are not recommended to guide patient therapy (*40*).

All Bcc isolates are intrinsically resistant to aminoglycosides (*40*), which we confirmed in our isolates. Intrinsic resistance also is described against chloramphenicol and tetracycline (*40*) (not tested) through the presence of efflux pumps. We identified several efflux pumps within the CH16 genome (*BSTAB16_5335–6*, _*4605–6*, and _*7210–1*), none of which are unique to the outbreak strain. Sensitivity to trimethoprim/sulfamethoxazole was a feature of the outbreak isolates; we did not identify trimethoprim/sulfamethoxazole resistance determinants in the draft genome.

Bcc is considered to be intrinsically and clinically resistant to many β-lactams through impermeability and the presence of inducible β-lactamases (*40*). All Bcc isolates tested were resistant to aminopenicillins, carboxypenicillins, and first-generation cephalosporins. Phenotypic resistance to third-generation cephalosporins, ureidopenicillins, and carbapenems was more variable among Bcc. We identified several β-lactamases in the CH16 genome, representing class A (*BSTAB16_4862* and _*4440*), class C (*BSTAB16_6957*), class D (*BSTAB16_5918*), and metallo-β-lactamases (*BSTAB16_ 3974* and _*5115*), none of which are unique to this strain.

The outbreak isolates are sensitive to ciprofloxacin, in contrast to *B. stabilis* BAA-67, with sporadic resistance seen among other Bcc isolates. Resistance can be associated with efflux (*40*) or specific mutations in *gyrA* (*BSTAB16_1445*). The *gyrA* of CH16 differs from that of strain BAA-67 at I83T and A700S (numbered according to *E. coli*). In general, this strain does not display enhanced antimicrobial resistance compared with other clinical Bcc isolates or *B. stabilis* BAA-67 (*2*,*41*,*42*).

Of note, some of the outbreak isolates had anomalous antibiograms, which we confirmed through repeated testing (Appendix 1 Table 7). This finding might relate to colony morphology because several morphotypes were observed during clinical work on the outbreak isolates. This phenomenon is known to occur within *Burkholderia* (*43*–*46*), resulting from reversible colony morphotype switching (*44*) or stable mutations (*43*,*46*). Altered genes often are involved in exopolysaccharide production, also causing changes in biofilm production, virulence, resistance, and motility (*43*–*46*), and might result from stress (*44*,*45*).

### Comparison of Outbreak Isolates

PFGE and k-mer analyses showed that the outbreak isolates cluster. We investigated how related the isolates are by comparing genomes using high-quality SNPs. The SNP phylogeny (Figure 2) indicates a maximum of 6 SNPs between isolates from the outbreak source, in agreement with Abdelbary et al. (*12*). The previously published core genome MLST (cgMLST) phylogeny used the same genome data but indicated up to 18 alleles difference between isolates (*4*), though these are likely artifacts of the methodology (*47*).

Washing glove isolates were located throughout the phylogeny; we observed diversity within a lot number (isolates E–F and L–O), and even within single packets (isolates A, B, and L–O). Patient samples also were found throughout the phylogeny, even those originating from the same city (isolates 5, 7, 9–13 are from a first city; 6, 8, 21, and 22 are from a second city; and 14–20 are from a third city), reflecting trends seen from cgMLST data (*4*).

SNP locations (Appendix 1 Table 8) indicate that, of the 40 nonsynonymous SNPs, 15 are in genes predicted to encode regulators, 6 in transporters, and 3 in flagellin. We could not tell whether these are random mutations or have been subject to selective pressure, but all classes are represented in both glove and patient isolates.

In addition to the SNPs, we saw some large-scale genomic differences. By mapping read data against each individual replicon, we noted that isolates E and 20 do not carry the plasmid, which appears to be a stochastic event because the SNP phylogeny indicates that these isolates are not derived from a common ancestor. Isolates D and E have highly similar PFGE patterns (Appendix 2 Figure 1), suggesting that the plasmid does not affect the PFGE results or was lost during laboratory culture. Patient isolate 22 also shows the loss of the first 52.5 kb of chromosome 3, representing an RD, which this isolate apparently lost spontaneously during the course of the outbreak. Because of this genome plasticity, we hypothesize the CH16 genome was changing even during the course of the outbreak.

## Discussion

We provide a thorough and detailed description of a *Burkholderia* sp. outbreak resolved by WGS (*4*) and illustrate various associated challenges, including morphotype differences, species designation, and a large genome with associated assembly, annotation, and interpretation issues. Defining species within Bcc is notoriously difficult (*26*), whether phenotypically by using biochemical or MALDI-TOF mass spectrometry profiles, genomically by using 16S rRNA gene sequences, or both. WGS provides the most thorough analysis and is increasingly cost and time effective, even compared with sequencing MLST loci, interpretation of which also is complex. We saw anomalies between the phenotype of this *B. stabilis* outbreak strain and those described in the literature (*26*,*27*) due to shorter than optimal test incubation times in standard clinical phenotyping (*29*).

Several techniques can be used on WGS data to provide phylogenies. K-mer analysis (Appendix 2 Figure 3) provides an indication of clustering, but the branch lengths cannot be relied on to provide a true phylogeny and do not truly reveal relationships within clades. With this technique, a lot of genomic information regarding the coding capacity of the genome is lost. cgMLST compares nucleotide sequences of CDSs common to a group of isolates, linking isolates with the highest numbers of identical alleles. During this process some genomic data necessarily are lost, with information from accessory genes and intergenic regions disregarded. However, both methods can be performed routinely with minimal training to enable rapid visualization of outbreak clusters. Comparing reads from all outbreak isolates to an assembled draft genome to generate a SNP phylogeny includes all genomic information but requires more computation, time, and expertise. 

WGS is the optimal way to determine the detailed relationships between isolates, giving insights into an outbreak and providing a basis from which to develop further typing methods. For future cases, we suggest rapid WGS, extraction of MLST alleles from assemblies for species identification as recommended by Mahenthiralingam et al. (*48*), and cgMLST typing for rapid outbreak identification. SNP detection can be a valuable subsequent step to determine accurate relatedness of isolates.

Bcc bacteria are known to survive in pharmaceutical and disinfectant materials (*1*,*48*,*49*). *B. stabilis* strains sharing MLST types can be isolated from the natural environment, hospitals, and patients (*50*), implicating the natural environment as a source of opportunistic *Burkholderia* and emphasizing the versatility of Bcc to survive and grow under diverse conditions. The CH16 genome displays features representative of *Burkholderia* in general; it is large, highly plastic, and contains many novel elements that might be involved in pathogenesis or environmental survival (*36*). The low number of pseudogenes and IS elements indicates that this strain has not undergone niche adaptation, and most likely is an opportunistic pathogen (*36*–*38*).

The cloud of diversity seen in the SNP phylogeny indicates that the source of the original contamination was not clonal or that several mutations occurred during the incubation of CH16 within the patient washing gloves. The loss of genomic elements, including the plasmid, from some of the isolates, also demonstrates the flexibility and the redundancy within such a large genome. Our study shows the importance of WGS in investigating and resolving this outbreak, which appears to have been caused by an environmental Bcc strain.

Appendix 1 Additional information on whole-genome sequencing and other analyses of *Burkholderia stabilis* strain CH16.

Appendix 2 Additional figures representing pulsed-field gel electrophoresis and whole-genome sequencing of *Burkholderia stabilis* strain CH16.
