# Supplementary material for: Phenotypic and Genomic Analyses of Burkholderia stabilis Clinical Contamination, Switzerland
Source: Emerg Infect Dis. 2019 Jun;25(6):1084–92. doi: 10.3201/eid2506.172119 (PMC6537712; doi:10.3201/eid2506.172119)
Supplement: Appendix 2 — Additional figures representing pulsed-field gel electrophoresis and whole-genome sequencing of Burkholderia stabilis strain CH16. [file 17-2119-Techapp-s2.pdf]

Article DOI: https://doi.org/10.3201/eid2506.172119

Phenotypic and Genomic Analyses of *Burkholderia stabilis* Clinical Contamination, Switzerland

Appendix 2

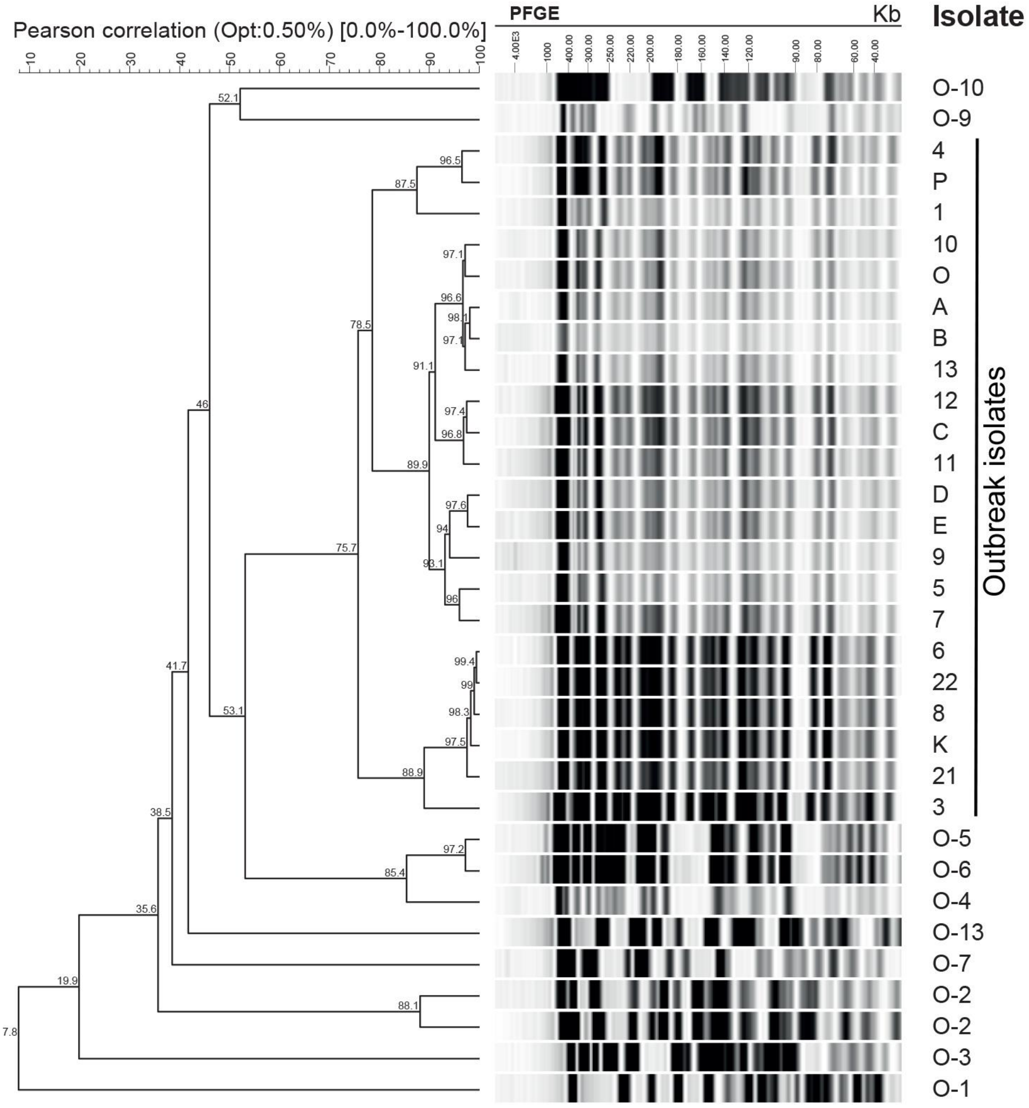

Appendix Figure 1. PFGE analysis of outbreak and unrelated strains of *Burkholderia stabilis*. Outbreak isolates form a cluster within the dendrogram (left); numbers indicate patient isolates, letters indicate isolates from washing gloves; relatedness is 75.7% using Pearson correlation analysis. Unrelated isolates, indicated with O- prefix, are clearly more distantly related. This method provided a relatedness of 88.1% for the isolate O-2 (*B. cepacia* ATCC25416) run in duplicate.

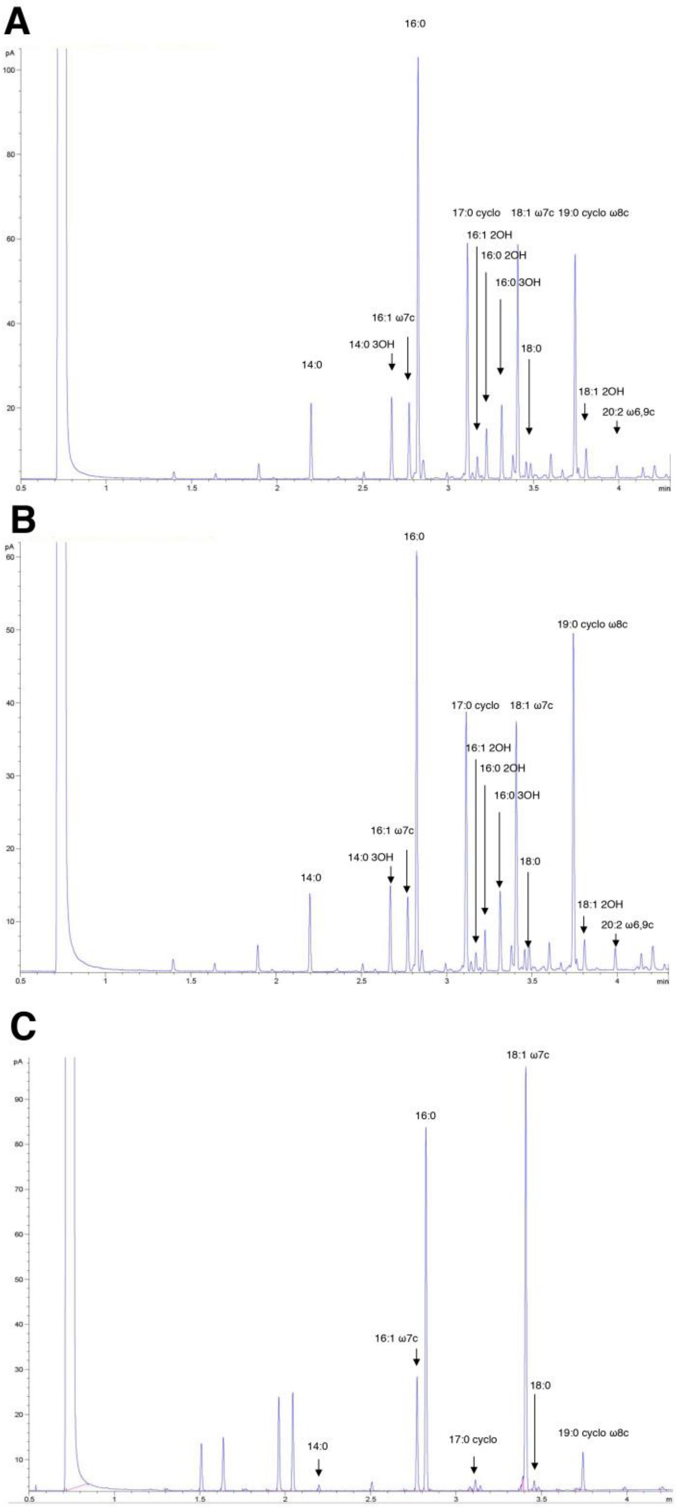

Appendix Figure 2. Gas chromatogram of the cellular fatty acids of outbreak and unrelated strains of *Burkholderia stabilis*. A) *B. stabilis* isolate 13, single measurement; B) *B. cepacia* unrelated clinical isolate O-6; C) unrelated isolate *P. aeruginosa* ATCC 27853. The outbreak strain has a cellular fatty acid profile that is easily recognized by the presence of 2 cyclopropane acids (17:0 cyclo [13.9 ± 0.4%], 19:0 cyclo ω8c [14.9 ± 2.2%]), 16:0 (23.7 ± 1.4%), and 18:1 ω7c (vaccenic acid; 13.4 ± 0.2%) as major acids, and 5 hydroxy acids 3-OH-14:0; 2-OH-16:1; 2-OH-16:0; 3-OH-16:0; and 2-OH-18:1.

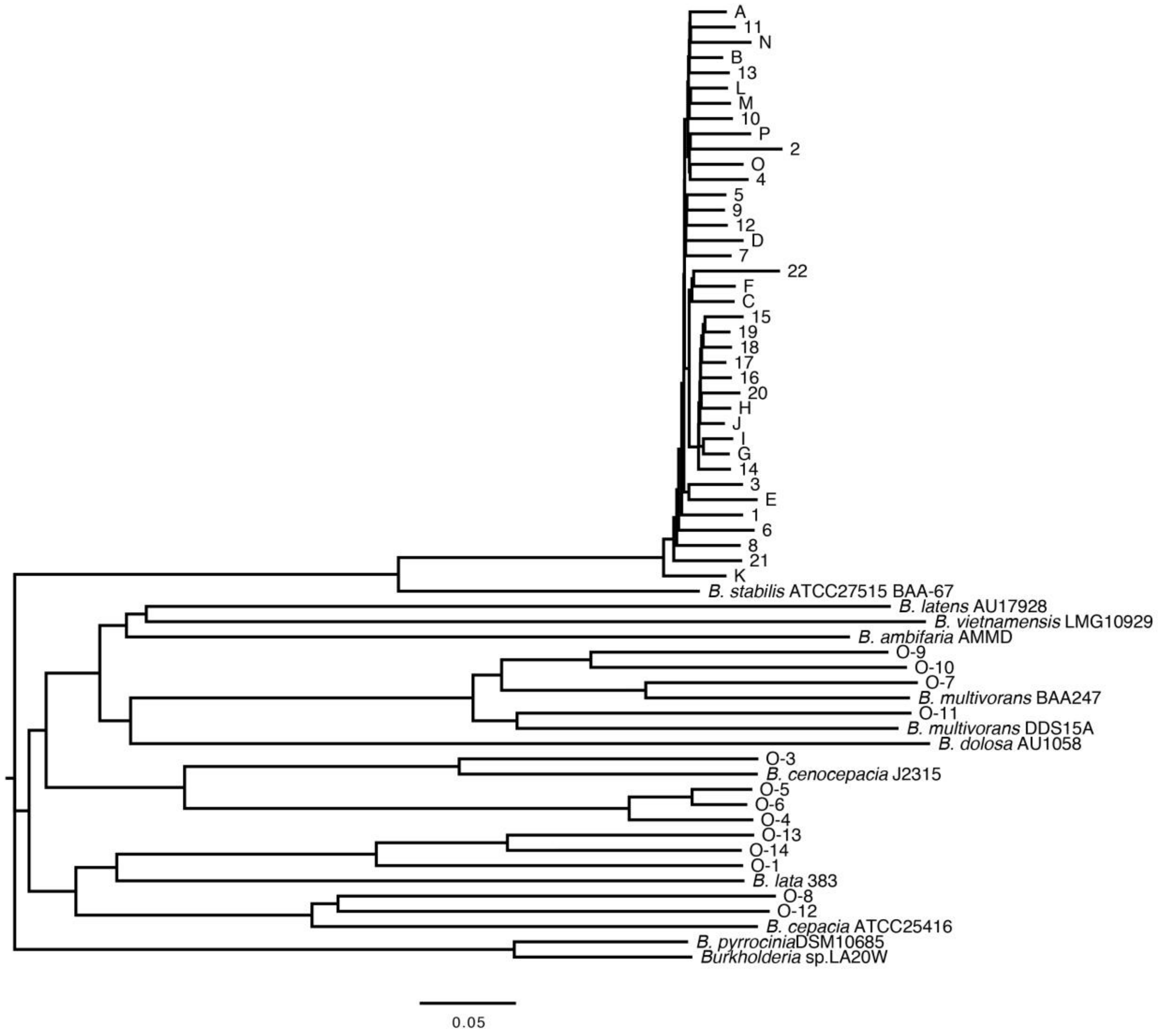

Appendix Figure 3. K-mer tree of *Burkholderia stabilis* outbreak isolates and controls. K-mer analysis was performed in CLC Genomics Workbench 9 (https://clc-genomics-workbench.software.informer.com/9.0) using default settings on read data from outbreak isolates, unrelated isolates, and published genomes of reference strains. The outbreak isolates clearly form a clade, most closely related to *B. stabilis* BAA-67. Scale bar indicates nucleotide substitutions per site.
